# Association between triglyceride glucose-body mass and one-year all-cause mortality of patients with heart failure: a retrospective study utilizing the MIMIC-IV database

**DOI:** 10.1186/s12933-023-02047-4

**Published:** 2023-11-08

**Authors:** Jiahao Dou, Chen Guo, Yawen Wang, Zihe Peng, Ruiyun Wu, Qiangqiang Li, Hong Zhao, Shoufang Song, Xuelu Sun, Jin Wei

**Affiliations:** 1 Department of Cardiology, The Second Affiliated Hospital of Xi’an Jiaotong University, Xiwulu 157#, Xi’an, Shaanxi 710004 China; 2 Clinical Research Center for Endemic Disease of Shaanxi Province, The Second Affiliated Hospital of Xi’an Jiaotong University, Xiwulu 157#, Xi’an, Shaanxi 710004 China; 3 Health Science Center, Xi’an Jiaotong University, 76 West Yanta Road, Xi’an, Shaanxi 710061 China

**Keywords:** Heart Failure, Triglyceride glucose-body mass (TyG-BMI), Insulin resistance, Prognosis, MIMIC-IV

## Abstract

**Background:**

The triglyceride glucose-body mass (TyG-BMI) index is acknowledged as both a reliable indicator of the risk of cardiovascular disease and an accurate surrogate biomarker for evaluating insulin resistance (IR). The importance of the TyG-BMI index among people with heart failure (HF), however, requires more investigation. The objective of this study was to inquire about the relationship between HF patients’ TyG-BMI index and their risk of 360-day mortality.

**Methods:**

The Medical Information Mart for Intensive Care (MIMIC-IV) database provided the study’s patient data, which were divided into quartiles according to their TyG-BMI index. The endpoint was mortality from all causes within 360 days. Kaplan-Meier analysis was used to compare this primary endpoint amongst the four groups indicated above. The association between the TyG-BMI index and the endpoint was investigated using restricted cubic splines and Cox proportional hazards analysis.

**Results:**

The study enrolled a total of 423 patients with HF (59.2% male), of whom 70 patients (16.9%) died within 360 days. Patients with higher TyG-BMI indexes had significantly lower mortality risks, according to the Kaplan-Meier analysis (log-rank P = 0.003). Furthermore, the restricted cubic spline analysis illustrated a decrease in the risk of all-cause mortality with an increasing TyG-BMI index. Additionally, multivariable Cox proportional hazards analyses showed that the risk of 360-day death from all causes was considerably higher in the lowest quartile of TyG-BMI. In comparison to the lowest TyG-BMI group, the fully adjusted Cox model yielded a hazard ratio (HR) of 0.24 (95% CI: 0.10, 0.59; p = 0.002) for 360-day mortality.

**Conclusions:**

In patients diagnosed with HF, a lower TyG-BMI index is strongly related to a higher risk of 360-day mortality. This index can be employed to categorize the risk levels of patients with HF and predict their one-year all-cause mortality .

**Supplementary Information:**

The online version contains supplementary material available at 10.1186/s12933-023-02047-4.

## Introduction

Heart failure (HF) presents a considerable medical challenge, impacting approximately 64 million individuals globally. Despite stable incidence rates globally, its mortality and incidence rates remain elevated, which imposes a substantial societal burden [1]. An important factor in the occurrence and development of HF is insulin resistance (IR). Likewise, HF can cause IR, contributing to a deleterious cycle [2, 3]. The significant advantages seen in HF patients who used Sodium-glucose co-transporter 2 (SGLT2) inhibitors imply that Diabetocardiology may be a focal point in the treatment of HF patients, deserving heightened attention [4].

Currently, there are no validated methods available for accurately determining insulin resistance (IR). The hyperinsulinemic-euglycemic clamp method, which is regarded as the most accurate method for determining insulin resistance, has drawbacks including complexity, high expense, and invasiveness [5]. Therefore, the homeostasis model assessment-estimated insulin resistance (HOMA-IR) index is extensively utilized currently. However, its applicability is restricted to individuals undergoing insulin treatment or those with non-functional beta cells [6]. The triglyceride-glucose (TyG) index, which has superiority in assessing IR in both patients with and without diabetes and applicability to all subjects regardless of their status as recipients of insulin treatment, was created to get over this limitation [7, 8]. More importantly, recent research has shown that the TyG index’s effectiveness in assessing IR can be significantly increased by combining it with obesity indicators including the waist-to-height ratio (WtHR), body mass index (BMI), and waist circumference (WC) [9, 10]. Other investigations discovered that the TyG-BMI index outperformed other indicators in assessing IR in the relevant association [11, 12]. Furthermore, Studies have demonstrated that the TyG-BMI index has a substantial correlation with a number of illnesses, including nonalcoholic fatty liver disease, hyperuricemia, diabetes mellitus (DM), and coronary heart disease (CHD) [13–16]. Nevertheless, the current literature lacks studies investigating the correlation between the TyG-BMI index and HF patients’ prognosis.

The study aims to elucidate the connection between the TyG-BMI index and the one-year all-cause mortality of HF patients. The findings of this study may help create fresh approaches for improving patient prognosis in this population while providing insightful information about the predictive value of the TyG-BMI index for HF patients’ clinical results.

## Methods

### Study participants

**Fig. 1:**
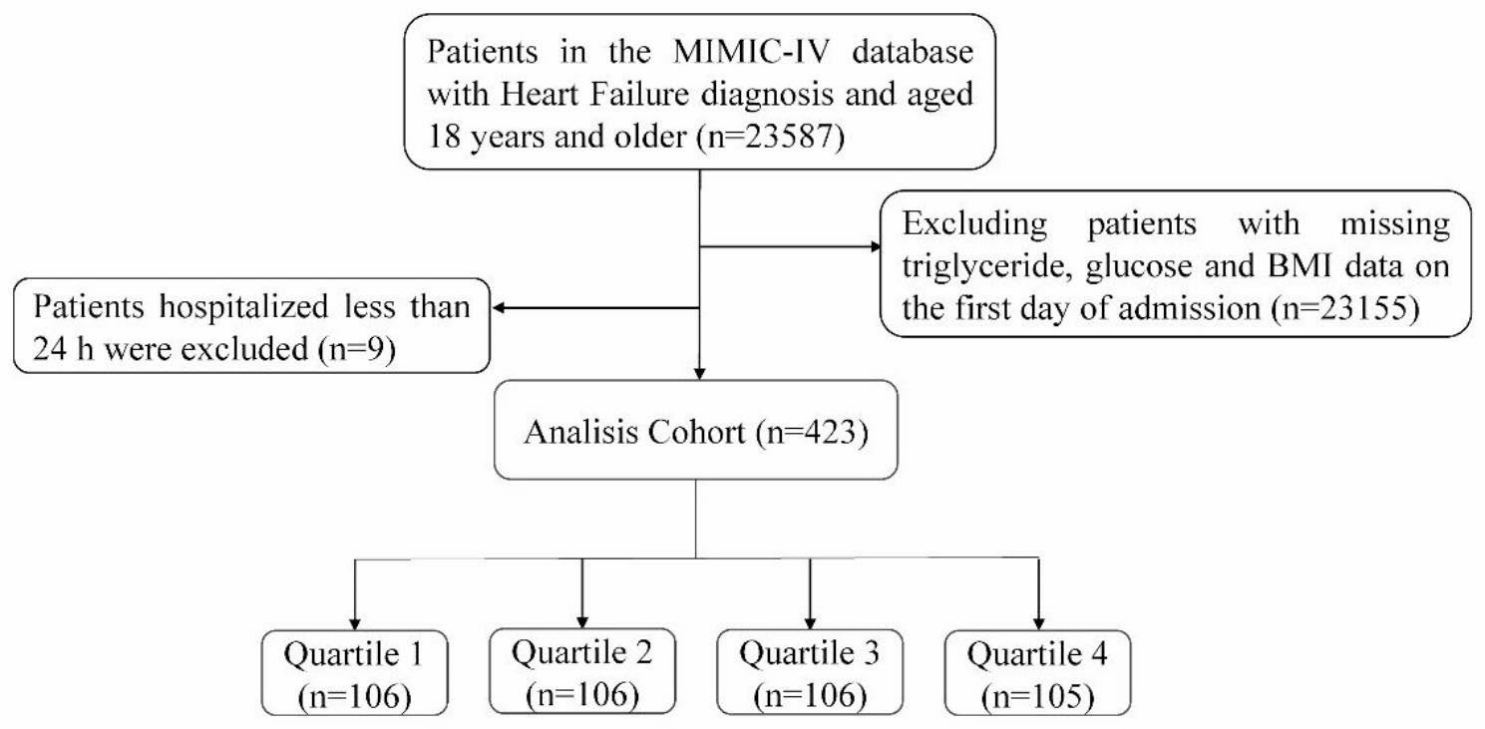
Flowchart of patient selection

The Massachusetts Institute of Technology (MIT) and Beth Israel Deaconess Medical Center (BIDMC) collaborated in developing the Medical Information Mart for Intensive Care (MIMIC-IV) electronic database (version 2.2), which was used in the present study. The database comprises pertinent information concerning patients who underwent inpatient treatment at BIDMC between 2008 and 2019. The Institutional Review Board (IRB) of BIDMC has waived informed consent and permitted the sharing of research resources since all data have undergone de-identification [17]. Prior to data extraction, the author Jiahao Dou fulfilled all the necessary requirements to access the database. The study included adult first-time hospitalized patients diagnosed with heart failure, based on the Ninth and Tenth Revisions of the International Classification of Diseases. The ICD codes for HF patients are listed in Additional file 2. Patients lacking data on admission day blood glucose, triglycerides, and BMI were excluded, as well as patients with a hospital stay shorter than 24 h. The flowchart for patient screening is presented in Fig. 1.

### Data extraction

The pgAdmin PostgreSQL tool (version 6.1) was used to extract information from the MIMIC-IV database, specifically from the following five categories: (1) Demographics, encompassing age, gender, race, marital status, and BMI; (2) Laboratory examination, including serum sodium, serum creatinine, aspartate aminotransferase (AST), red blood cell distribution width (RDW), white blood cell (WBC), red blood cell (RBC), hemoglobin, hematocrit, glucose, glycated hemoglobin A1c (HbA1c), total cholesterol (TC), low-density lipoprotein cholesterol (LDL-C), triglyceride (TG), and N-terminal pro-B-type natriuretic peptide (NT-proBNP), C-reactive protein (CRP) ; (3) Comorbidities, such as CHD, atrial fibrillation (AF), DM, chronic obstructive pulmonary disease (COPD), and chronic kidney disease (CKD); (4) Medication treatments, consisting of diuretics, beta-blockers, and angiotensin-converting enzyme inhibitor or angiotensin receptor blocker (ACEI/ARB); and (5) Duration of hospitalization, follow-up survival status, and follow-up survival time (with the database offering 1-year follow-up information for all discharged patients). The TyG-BMI index was computed according to the TG, BMI and fasting blood glucose (FBG) concentrations according to the equation: ln [TG (mg/dL) × FBG (mg/dL)/2] × BMI [18]. All laboratory variables were exclusively obtained from the initial 24 h after patient admission, and in cases of multiple results, the average value was utilized. Variables with missing values exceeding 20% were excluded to mitigate potential bias. Variables with less than 20% missing values were imputed using the random forest imputation method implemented in the missForest package of R software [19].

### Outcomes

The main objective of this study was 360-day all-cause mortality. Secondary outcomes focused on 28-day all-cause mortality.

### Statistical analysis

Statistics were conducted using t-tests or analysis of variance (ANOVA), and constant variables were reported as the mean ± standard deviation, or median (interquartile range, IQR). For categorical variables, numbers (proportions) were employed for presentation, and their analysis was carried out by means of chi-square tests, corrected chi-square tests, or Fisher’s exact test. Kaplan-Meier survival analysis was employed to evaluate the incidence rate of major outcome events across distinct stratified groups based on the TyG-BMI index. Log-rank tests were utilized to assess any observed disparities. Univariable Cox regression analysis was performed to examine the association between the TyG-BMI index and the mortality rate at 360 days. Variables with clinical significance and a significance level of P < 0.05 were incorporated into the multivariable Cox proportional hazards model. The model 1 solely included the TyG-BMI index without any further adjustments. In Model 2, adjustments were made for age, gender, and race. Model 3 incorporated further adjustments for laboratory tests and comorbidities such as LDL-C, HDL-C, COPD, CKD. The reference category for all models was the lowest quartile of the TyG-BMI index. We assessed the predictive ability, sensitivity, and specificity of the TyG-BMI index for predicting the primary outcome by employing a receiver-operating characteristic (ROC) curve analysis. We used a restricted cubic spline analysis to capture the dose-effect relationship connection with the TyG-BMI index and the risk of the primary result. Furthermore, we performed stratified analyses to evaluate the consistency of the TyG-BMI index’s prognostic values with the primary outcome. The analyses took into account gender, age (≤ 68 years and > 68 years), as well as the presence of comorbidities such as DM, CHD, and AF. The data analyses were conducted using R software (version 4.2.2) and SPSS statistical software (Version 26.0). For all analyses, a two-sided P-value below 0.05 was considered statistically significant.

## Results

### Baseline characteristics

Based on the quartiles of the TyG-BMI index, Table 1 (in Additional file 1) lists the baseline characteristics for HF patients. The participants had a mean age of 68 years, with 59.2% of them being male. Patients in the higher TyG-BMI index group were generally younger and had elevated levels of RBC, Platelet, Hemoglobin, HbA1c, TC, and HCT, as well as lower levels of HDL and NT-proBNP. Furthermore, they exhibited a higher prevalence of DM but a lower prevalence of AF compared to the lower group. Additionally, the higher TyG-BMI group had an increased use of diuretics (all p < 0.05). With increasing TyG-BMI index, 360 days mortality (25.5% vs. 19.8% vs. 12.3% vs. 8.5%, P = 0.004) decreased gradually. However, no significant differences were observed in 28-day mortality (p = 0.090).

### Primary outcomes

**Fig. 2:**
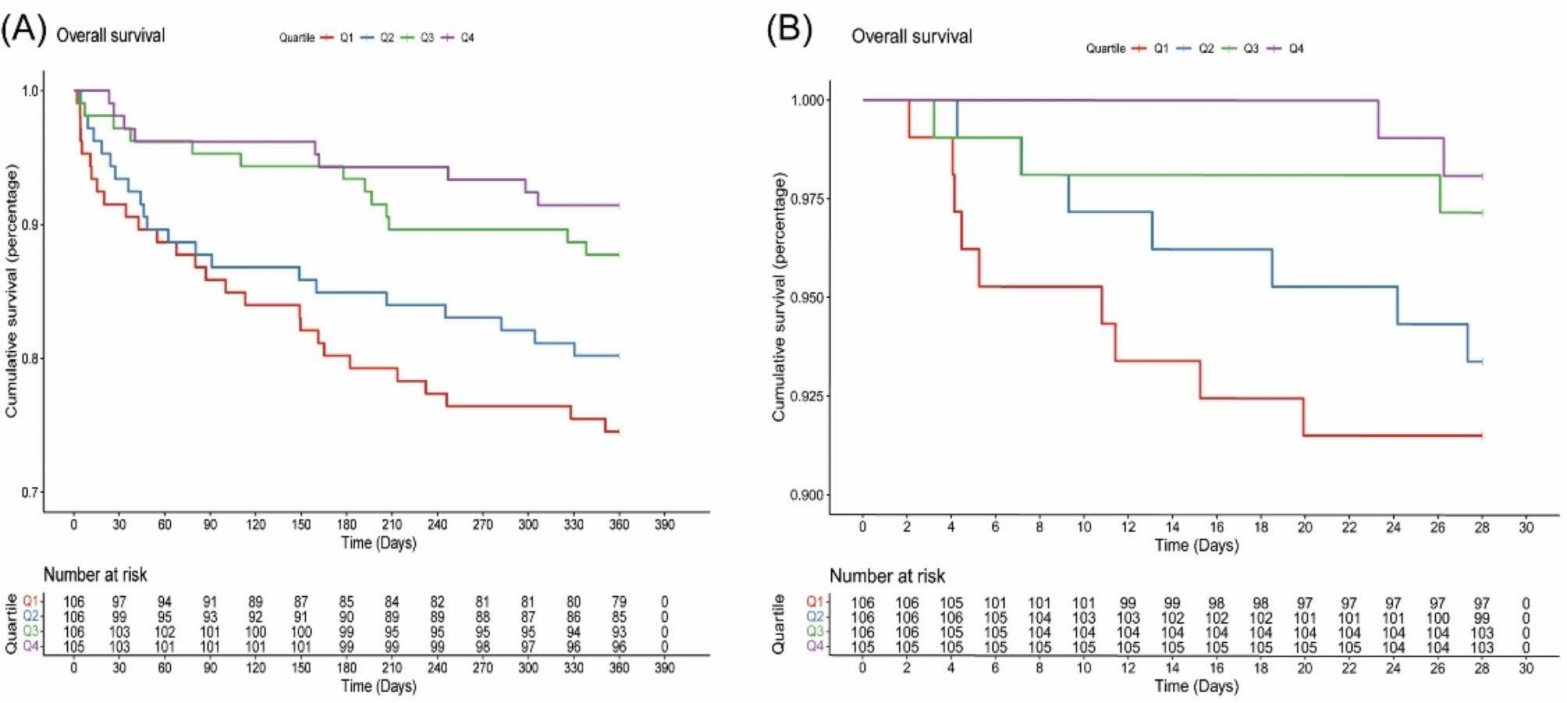
Kaplan–Meier survival analysis curves for all-cause mortality TyG-BMI index: Q1 (44.30–209.09), Q2 (209.09–254.27), Q3 (254.27–314.09), Q4 (314.09–575.70). Kaplan–Meier curves showing the cumulative probability of all-cause mortality according to groups at 28-day (**A**), and 360-day (**B**)

The Kaplan-Meier survival analysis curves in Fig. 2 show the prevalence of all-cause mortality in several groups that have been divided based on the TyG-BMI index quartiles.

**Fig. 3:**
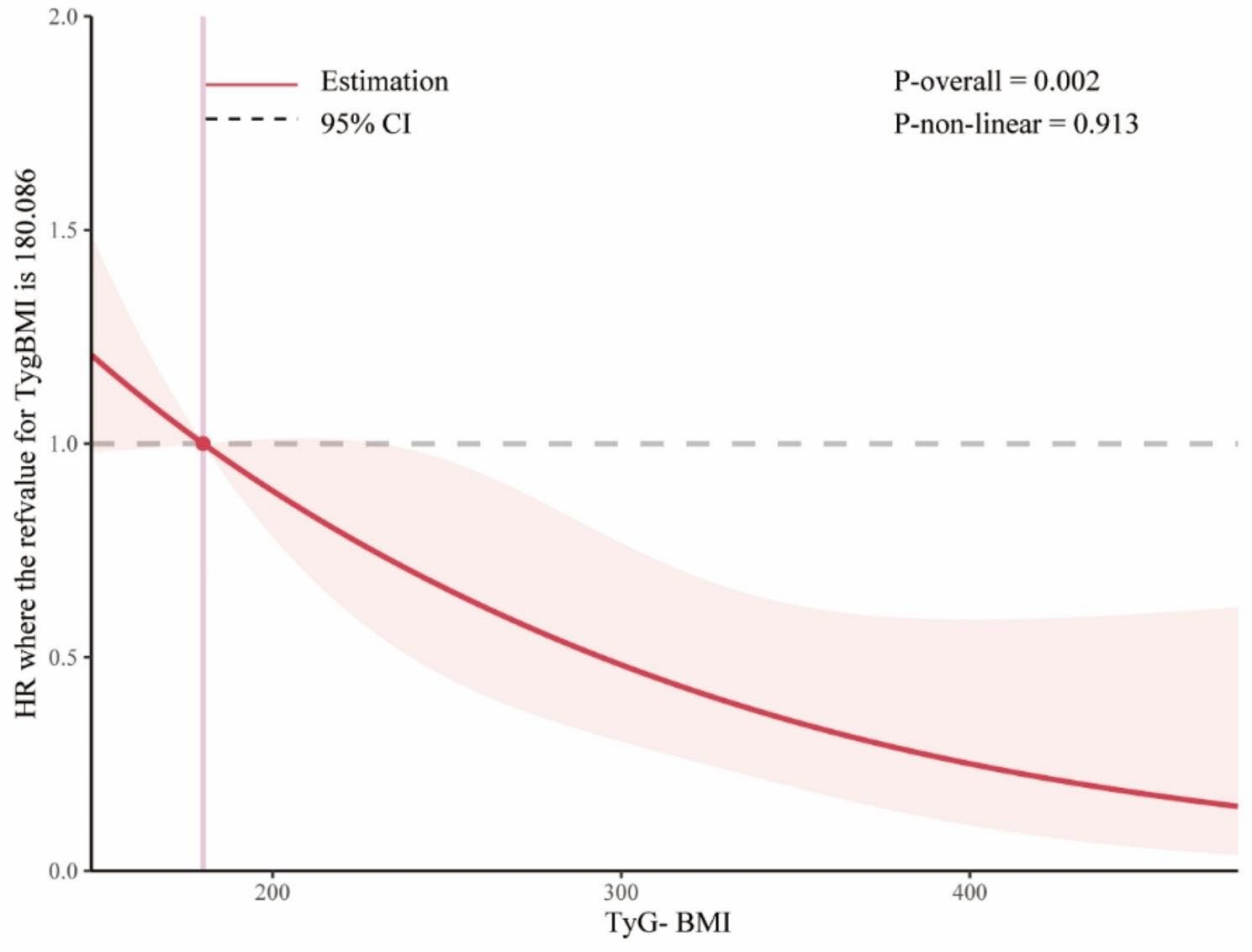
Restricted cubic spline regression analysis of TyG-BMI index with in all-cause mortality Abbreviation :TyG-BMI index: triglyceride glucose-body mass index

Patients with a higher TyG-BMI index demonstrated a significantly higher 360-day survival rate compared to those with a lower index (log-rank P = 0.003). In contrast, there was no significant difference observed in the 28-day timeframe (log-rank P = 0.085). The restricted cubic spline (RCS) model revealed an L-shaped connection between the index and the all-cause death rate over a 360-day period using the TyG-BMI index as a constant variable (Fig. 3).

**Fig. 4:**
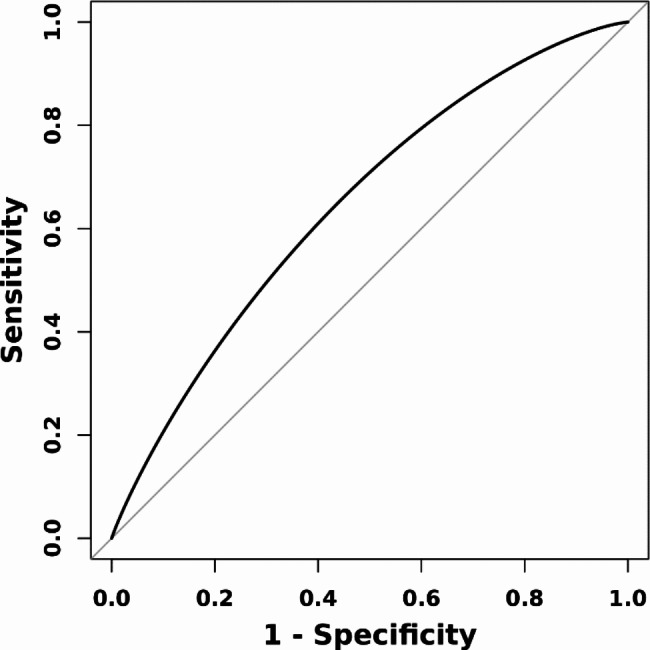
ROC curve assesses the predictive capability of the TyG-BMI index for all-cause mortality Abbreviation: ROC, receiver-operating characteristic; TyG-BMI, triglyceride glucose-body mass index

The inflection point of the RCS curve was determined to be at TyG-BMI = 289. Based on this inflection point, the data were divided into two groups. Segmented regression analysis was conducted on each group individually, presenting the outcomes as follows:

HR = 0.73 (95% CI 0.57–0.92, P = 0.009) for TyG-BMI < 289, and HR = 0.52 (95% CI 0.25–1.07, P = 0.075) for TyG-BMI ≥ 289. The ROC curve revealed a moderate ability of TyG-BMI to predict 360 days mortality (AUC = 0.647 (0.581, 0.715), the ideal cut-off point was 244.35, the specificity was 59.2%, and the sensitivity was 67.1% (Fig. 4).

When categorizing the TyG-BMI quartiles, patients in the higher TyG-BMI quartile were significantly associated with a higher 360-day survival rate in the three established Cox proportional hazards models that were constructed and exhibited an increasing trend with higher TyG-BMI indices(Table 2 in Additional file 1).

### Subgroup analysis

The subgroup analysis demonstrated consistent associations among the TyG-BMI index quartiles and risks of primary results in various subgroups, such as sex, marital status, AF, CHD, COPD, hyperlipidemia, and the usage of statins, diuretics, antiplatelet medications, and ACEI/ARB (Table 3 in Additional file 1). The relationship between TyG and risk of 360-day mortality was consistent among male patients, married patients, those diagnosed with AF, hyperlipidemia, without CHD, COPD, and individuals using statins, diuretics, and antiplatelet medications, but not ACEI/ARB patients. It is worth noting that similar associations exist in both patients with and without DM or CKD. Additionally, no significant interactions between the factors in the subgroup examination and the TyG-BMI index were found (all p-values for interaction > 0.05).

## Discussion

The TyG-BMI index and mortality from all causes in patients with HF were investigated for the first time in this study, which found a strong inverse association between the two variables. Despite adjusting for a wide variety of confounding factors, higher TyG-BMI index levels are significantly correlated with lower mortality rates, and this conclusion remains consistent. This correlation may be attributed to the “obesity paradox”.

IR refers to the reduced or impaired responsiveness of insulin-dependent organs and tissues to both endogenous and exogenous insulin, which has a close relationship between the occurrence and development of HF [3]. On one hand, IR can cause HF. In a 9-year prospective study conducted in Sweden, involving nearly 12,000 participants without HF, it was discovered that insulin IR precedes HF and acts as an independent risk factor, regardless of other risk factors such as diabetes [20]. Held et al. demonstrated that for patients with diabetes, each rise of one millimole per liter in fasting blood glucose is related to a 1.10. times greater risk of hospitalization due to congestive heart failure [21]. The specific mechanisms include: reduced capacity for myocardial energy metabolism and disorder in substrate metabolism, abnormal calcium signaling pathway, and impaired NO signaling pathway between myocardial cells and endothelial cells [22–26]. On the other hand, HF can also cause IR. According to the findings of the Leibniz Institute for Prevention Research and Epidemiology (BIPS), individuals with HF have a 13% increased likelihood of having diabetes compared to the general population [27]. Research has shown that patients with HF not only have systemic IR but also often manifest myocardial IR [28, 29]. The mechanisms include overactive stimulation of the renin-angiotensin-aldosterone system (RAAS) and the sympathetic nervous system (SNS), as well as imbalanced secretion of cytokines [30, 31].

Undoubtedly, IR contributes significantly as a risk variable to the onset and progression of HF. The value and accuracy of the TyG-BMI index in assessing IR also have been validated in numerous studies. The relationship between TyG index and heart failure has been confirmed by multiple studies. Multiple studies have demonstrated a significant association between a high TyG index and an increased risk of heart failure, mortality, and readmission in patients with heart failure. Moreover, relevant studies have consistently reported the excellent diagnostic and predictive capabilities of the TyG index [32–34]. Therefore, the “obesity paradox” may be the explanation for the negative correlation between the TyG-BMI index and all-cause mortality in HF patients.

Although a greater BMI is linked to a higher risk of developing heart failure, the connection between BMI and outlook in those who have been diagnosed with HF is unexpectedly complex [35]. Scientific literature on HF has extensively shown the association between this prognosis and conventional risk factors [36]. Higher BMI categories were strongly linked with a decreased risk of death, according to a massive random control trial comprising 7599 patients with HF [37]. Furthermore, a substantial finding was also found in research examining the relationship between BMI and death in the hospital in a sample of 108,927 patients with decompensated heart failure.For each 5-unit increment in BMI, the results showed a 10% reduction in the mortality (P < 0.001) [38]. With a total of 22,807 participants, a meta-analysis was performed aimed at looking at the connection between BMI and outcomes for those with HF. Findings demonstrated that individuals with a low BMI were at the highest risk for HF hospitalizations and all-cause mortality, while those classified as overweight had the lowest risk [39].

The “obesity paradox” in HF can be attributed to the following predominant mechanisms. Firstly, patients with HF are generally in a catabolic state, and obesity or overweight may suggest a greater presence of physiological reserves and can potentially lead to a more favorable prognosis [36]. Secondly, B-natriuretic peptide levels are frequently decreased in obese people, demonstrating that this specific subset of people has better hemodynamic properties [40]. These patients are capable of maintaining elevated blood pressures, potentially preserving renal function and allowing individuals to endure more beneficial medications for prognosis such as neprilysin inhibitors, angiotensin receptor blockers, angiotensin-converting enzyme inhibitors (ACEI), and beta-blockers [41]. Thirdly, those patients may derive advantages from the preventative properties that anti-inflammatory adipokines exert. In HF, elevated amounts of tumor necrosis factor-alpha (TNF-α) may induce heart damage due to its pro-apoptotic and detrimental impact on the myocardium. Nevertheless, adipose tissue generates soluble TNF-α receptors that demonstrate cardioprotective properties by counteracting the biological actions of TNF-α [42, 43]. Additionally, obesity is linked to reduced levels of adiponectin, an adipokine that enhances energy expenditure and promotes weight loss. However, these effects are detrimental for HF patients in a catabolic state [44]. Finally, the presence of adipose tissue is frequently linked to elevated muscular strength and enhanced cardiopulmonary function in HF patients, which is advantageous for them [45]. The research findings have significant implications for clinical practice and patient self-management. Firstly, TyG-BMI index can serve as an effective predictive indicator in the management of HF patients. Patients who have lower TyG-BMI index experience an increased probability of death. Secondly, paying close attention to the BMI and nutritional status of HF patients is highly significant, and providing early nutritional support is vital for those with low body weight or poor nutritional status. Furthermore, gastrointestinal edema in HF patients may result in reduced appetite and weight loss, which might manifest before obvious symptoms like limb edema and breathing difficulties. The survival rate of patients with HF may be increased by the prompt identification and management of these symptoms.

But there were certain restrictions on our study as well. Firstly, this retrospective investigation depends on observational research, which limits the ability to establish a definitive causal relationship. Although we utilized multivariable adjustments and subgroup analysis, potential confounders may still affect the clinical prognosis. For instance, cardiac ultrasound data was not publicly accessible in the most recent version of the database, while variables like NT-proBNP and APACHE scores were not considered due to a significant amount of missing data. Furthermore, given the database limitations, it is not possible to ascertain whether all glucose and lipid measurements were obtained from fasting individuals. Thirdly, this study exclusively focused on evaluating the baseline TyG-BMI index. Further investigation is required to determine the prognostic significance of dynamic changes in the TyG-BMI index during hospitalization and follow-up.Fourthly, we were unable to compare the TyG-BMI index with other widely recognized IR measuring methods due to the absence of relevant information in the database. Finally, this study had a sample size of moderate magnitude, a relatively short follow-up duration, and was solely conducted at a single center, potentially introducing selection bias. Future research should diligently address the aforementioned issues to the fullest extent possible, with the aim of providing robust evidence to bolster our findings.

## Conclusion

In spite of additional risk variables, we found that the TyG-BMI index indicated a better one-year all-cause mortality outcome for those with HF. The tyG-BMI index may serve as an effective tool for classifying and treating the risks associated with HF patients. Moreover, for patients diagnosed with heart failure, BMI should receive attention as it significantly affects prognosis.

### Electronic supplementary material

Below is the link to the electronic supplementary material.

Supplementary Material 1

Supplementary Material 2

## Data Availability

The corresponding author can be contacted to receive the datasets generated and utilized in this work upon reasonable request and with MIMIC’s permission.

## Abbreviations

ACEI/ARB: Angiotensin-converting enzyme inhibitor or angiotensin receptor blocker
AF: Atrial fibrillation
BIDMC: Beth Israel Deaconess Medical Center
BIPS: Leibniz Institute for Prevention Research and Epidemiology
BMI: Body mass index
CHD: Coronary heart disease
CKD: Chronic kidney disease
COPD: Chronic obstructive pulmonary disease
CRP: C-reactive protein
DM: Diabetes mellitus
HbA1c: Glycated hemoglobin A1c
HF: Heart failure
HOMA-IR: Homeostasis model assessment-estimated insulin resistance
IR: Insulin resistance
IRB: Institutional Review Board
LDL-C: Low-density lipoprotein cholesterol
MIMIC-IV: Medical Information Mart for Intensive Care
MIT: Massachusetts Institute of Technology
NT-proBNP: N-terminal pro-B-type natriuretic peptide
RAAS: Renin-angiotensin-aldosterone system
RBC: Red blood cell
RCS: Restricted cubic spline
ROC: Receiver-operating characteristic
SGLT2i: Sodium-glucose co-transporter 2 inhibitors
SNS: Sympathetic nervous system
TC: Total cholesterol
TG: Triglyceride
TNF-α: Tumor necrosis factor-alpha
TyG-BMI index: Triglyceride glucose-body mass index
TyG: Triglyceride-glucose
WBC: White blood cell
WC: Waist circumference
WtHR: Waist-to-height ratio
