# Supplementary material for: Association between triglyceride glucose-body mass and one-year all-cause mortality of patients with heart failure: a retrospective study utilizing the MIMIC-IV database
Source: Cardiovasc Diabetol. 2023 Nov 8;22:309. doi: 10.1186/s12933-023-02047-4 (PMC10634170; doi:10.1186/s12933-023-02047-4)
Supplement: Supplementary file 1 — Supplementary Material 1 [file 12933_2023_2047_MOESM1_ESM.docx]

Table 1 Baseline characteristics of Heart Failure patients grouped according to TyG-BMI index quartile

| Categories | Overall  (N=423) | Q1  (N=106) | Q2  (N=106) | Q3  (N=106) | Q4  (N=105) | P-value |  |  |
| --- | --- | --- | --- | --- | --- | --- | --- | --- |
| Demographic |  | | | | | |  |  |
| Age, years, mean (SD) | 67.98(15.30) | 72.54(13.36) | 71.75(14.98) | 66.33(14.92) | 61.31(15.32) | <0.001 |  |  |
| Male,n(%) | 251(59.2) | 52(49.1) | 66(62.3) | 64(60.4) | 69(65.1) | 0.089 |  |  |
| Ethnicity,n(%) | 0.019 | | | | | |  |  |
| Asian | 12（2.83） | 7（6.60） | 3（2.83） | 1（0.94） | 1（0.94） |  |  |  |
| White | 286（67.45） | 68（64.15） | 83（78.30） | 72(67.92) | 63(59.43) |  |  |  |
| Black | 49（11.56） | 12（11.32） | 5（4.72） | 12（11.32） | 20(18.87) |  |  |  |
| Hispanic/Latino | 19（4.48） | 3（2.83） | 2（1.89） | 7(6.60) | 7(6.67) |  |  |  |
| Other | 57（13.44） | 16（15.09） | 13（12.26） | 14(13.21) | 14(13.33) |  |  |  |
| BMI,kg/m^2^, mean (SD) | 30.62(8.67) | 21.55 (3.26) | 26.76 (2.23) | 32.28 (2.97) | 42.02(6.89) | <0.001 |  |  |
| Marital status(%) | 0.015 | | | | | |  |  |
| Married | 184(43.50) | 34(32.08) | 56(52.83) | 48(45.28) | 46(43.81) |  |  |  |
| Single | 34(8.04) | 7(6.60) | 7(6.60) | 10(9.43) | 10(9.52) |  |  |  |
| Divorced | 120(28.37) | 37(34.91) | 18(16.98) | 30(28.30) | 35(33.33) |  |  |  |
| other | 85(20.10) | 28(23.58) | 25(23.54) | 18(16.98) | 14(13.33) |  |  |  |
| Laboratory tests |  | | | | | |  |  |
| RBC,m/µL, mean (SD) | 4.05 (0.77) | 3.77 (0.76) | 4.05 (0.75) | 4.07 (0.73) | 4.30 (0.77) | <0.001 |  |  |
| WBC, K/µL, mean (SD) | 8.79 (10.16) | 9.61 (19.88) | 8.12 (2.91) | 8.91 (3.52) | 8.56 (2.44) | 0.758 |  |  |
| Platelet,K/µL,mean(SD) | 218.13 (84.93) | 201.06 (94.76) | 213.64 (84.59) | 234.33 (81.97) | 223.03 (74.94) | 0.036 |  |  |
| Hemoglobin,g/dL,mean(SD) | 11.86 (2.23) | 11.31 (2.21) | 12.10 (2.21) | 11.76 (2.09) | 12.27 (2.33) | 0.012 |  |  |
| Glucose, mg/dL, mean (SD) | 125.22 (47.31) | 106.53 (32.13) | 123.22 (47.31) | 122.25 (36.10) | 148.88 (59.27) | <0.001 |  |  |
| HbA1c,%, mean (SD) | 6.45 (1.61) | 5.75 (0.93) | 6.27 (1.42) | 6.35 (1.24) | 7.30 (2.12) | <0.001 |  |  |
| TG, mg/dL, mean (SD) | 127.99 (115.86) | 86.60 (47.55) | 104.16 (52.27) | 133.01 (109.52) | 188.18 (176.36) | <0.001 |  |  |
| LDL, mg/dL, mean (SD) | 84.72 (38.37) | 79.02 (34.78) | 80.56 (35.27) | 87.22 (42.95) | 91.71 (39.06) | 0.065 |  |  |
| HDL,mg/dL, mean (SD) | 45.02 (16.16) | 51.75 (16.72) | 49.00 (16.72) | 39.90 (13.13) | 39.79 (14.39) | <0.001 |  |  |
| TC, mg/dL, mean (SD) | 153.93 (49.94) | 147.80 (42.50) | 148.50 (43.02) | 153.85 (61.55) | 165.08 (49.27) | 0.047 |  |  |
| TC/HDL,mean(SD) | 3.79 (1.69) | 3.11 (1.54) | 3.34 (1.28) | 4.17 (1.85) | 4.51 (1.66) | <0.001 |  |  |
| NT-proBNP,  pg/mL,mean(SD) | 5486.97 (6926.76) | 7676.82 (7880.91) | 6704.37  (7599.71) | 5803.78  (7626.27) | 2938.83  (3784.81) | 0.001 |  |  |
| cTnT, ng/mean(SD) | 0.47 (0.99) | 0.45 (0.95) | 0.64 (1.35) | 0.45 (0.72) | 0.32 (0.72) | 0.432 |  |  |
| CK-MB, ng/ml, mean(SD) | 10.73 (28.71) | 12.71 (36.18) | 12.49 (30.51) | 6.44 (10.71) | 10.81 (29.37) | 0.591 |  |  |
| Creatinine, mg/dL, mean(SD) | 1.39 (1.28) | 1.33 (1.30) | 1.39 (1.40) | 1.57 (1.59) | 1.25 (0.58) | 0.302 |  |  |
| BUN,mg/dL,mean(SD) | 25.53 (16.39) | 25.87 (15.33) | 25.12 (16.28) | 27.12 (19.53) | 24.02 (14.01) | 0.570 |  |  |
| AST,IU/L, mean(SD | 58.79 (206.29) | 70.42 (276.16) | 57.35 (152.52) | 46.26 (82.78) | 62.10 (259.58) | 0.887 |  |  |
| ALT,IU/L, mean(SD | 54.26 (225.57) | 55.16 (201.03) | 52.09 (170.35) | 41.95 (77.30) | 68.50 (361.37) | 0.889 |  |  |
| Albumin,g/dL, mean (SD) | 36.86 (5.12) | 35.83 (5.24) | 37.21 (4.66) | 36.90 (5.60) | 37.54 (4.82) | 0.215 |  |  |
| Sodium,mEq/L,mean(SD) | 139.78 (3.42) | 140.14 (3.30) | 139.05 (3.99) | 139.90 (3.12) | 140.01 (3.13) | 0.081 |  | |
| Potassium,mEq/L, mean (SD) | 4.15 (0.47) | 4.11 (0.44) | 4.18 (0.48) | 4.16 (0.51) | 4.17 (0.44) | 0.756 |  |  |
| Calcium, mg/dl, mean (SD) | 9.01 (0.53) | 8.95 (0.59) | 9.00 (0.45) | 8.99 (0.57) | 9.09 (0.49) | 0.320 |  |  |
| Magnesium, mg/dl, mean(SD) | 2.01 (0.24) | 2.00 (0.29) | 2.00 (0.20) | 2.03 (0.26) | 2.01 (0.20) | 0.823 |  |  |
| RDW | 14.90 (2.30) | 15.22 (2.66) | 14.74 (2.32) | 14.63 (1.72) | 15.02 (2.38) | 0.240 |  |  |
| RDW-SD | 49.08 (8.34) | 51.45 (10.58) | 49.54 (7.99) | 47.56 (5.78) | 48.12 (8.36) | 0.014 |  |  |
| Hematocrit | 36.62 (6.41) | 34.95 (6.48) | 37.07 (6.34) | 36.45 (6.02) | 37.98 (6.50) | 0.007 |  |  |
| TyG index | 8.74 (0.72) | 8.30 (0.50) | 8.61 (0.59) | 8.81 (0.60) | 9.25 (0.79) | <0.001 |  |  |
| TyG-BMI index | 269.17(84.92) | 178.36 (26.04) | 229.43 (13.72) | 282.96 (17.81) | 387.08 (61.74) | <0.001 |  |  |
| Comorbidities |  | | | | | |  |  |
| Hypertension,n,% | 77 (18.2) | 24 (22.6) | 19 (17.9) | 20 (18.9) | 14 (13.2) | 0.284 |  |  |
| Diabetes,n,% | 163 (38.4) | 19 (17.9) | 35 (33.0) | 45 (42.5) | 64 (60.4) | <0.001 |  |  |
| CHD, n (%) | 178(42.0) | 38(35.8) | 54(50.9) | 44(41.5) | 42(39.6) | 0.151 |  |  |
| AF, n (%) | 166 (39.2) | 49 (46.2) | 48 (45.3) | 40 (37.7) | 29 (27.4) | 0.012 |  |  |
| COPD, n (%) | 51 (12.0) | 12 (11.3) | 16 (15.1) | 10 (9.4) | 13 (12.3) | 0.643 |  |  |
| Hyperlipidemia,n,% | 220 (51.9) | 51 (48.1) | 60 (56.6) | 54 (50.9) | 55 (51.9) | 0.661 |  |  |
| CKD,n,(%) | 124 (29.3) | 29 (27.4) | 29 (27.4) | 33 (31.1) | 33 (31.4) | 0.853 |  |  |
| Medicine |  | | | | | |  |  |
| Diuretic | 316 (74.5) | 70 (66.0) | 74 (69.8) | 81 (76.4) | 91 (85.8) | 0.006 |  |  |
| ACEI/ARB | 233 (55.0) | 50 (47.2) | 57 (53.8) | 65 (61.3) | 61 (57.5) | 0.197 |  |  |
| Beta-blocker | 334 (78.8) | 77 (72.6) | 87 (82.1) | 83 (78.3) | 87 (82.1) | 0.286 |  |  |
| Antiplatelet | 321 (75.7) | 80 (75.5) | 83 (78.3) | 78 (73.6) | 80 (75.5) | 0.884 |  |  |
| Statin | 305 (71.9) | 74 (69.8) | 81 (76.4) | 75 (70.8) | 75 (70.8) | 0.697 |  |  |
| Digoxin | 34 (8.0) | 11 (10.4) | 9 (8.5) | 7 (6.6) | 7 (6.7) | 0.771 |  |  |
| Events |  | | | | | |  |  |
| 28 days death,n,(%) | 21 (4.9) | 9(8.5) | 7 (6.6) | 3 (2.8) | 2(1.9) | 0.090 |  |  |
| 360 days death,n,(%) | 70 (16.5) | 27 (25.5) | 21 (19.8) | 13 (12.3) | 9 (8.5) | 0.004 |  |  |
|  |  |  |  |  |  |  |  |  |

TyG-BMI index: Q1 (44.30–209.09), Q2 (209.09–254.27), Q3 (254.27–314.09), Q4 (314.09–575.70)

TyG-BMI index triglyceride glucose-body mass index, BMI body mass index, RBC red blood cell, WBC white blood cell, HbA1c hemoglobin A1c, TC total cholesterol, TG triglyceride, LDL low-density lipoprotein, HDL high-density lipoprotein, TC/HDL total cholesterol/ high-density lipoprotein ratio, NT-proBNP N-terminal pro-B-type natriuretic peptide, cTnT cardiac troponins T, CK-MB creatine kinase isoenzymes, BUN blood urea nitrogen, ALT alanine aminotransferase, AST aspartate aminotransferase, RDW red blood cell distribution width, RDW SD red blood cell distribution width-standard deviation, TyG triglyceride-glucose, CHD coronary heart disease, AF atrial fibrillation, COPD chronic obstructive pulmonary disease, CKD chronic kidney disease, ACEI/ARB angiotensin-converting enzyme inhibitor or angiotensin receptor blocker

Table 2. Cox proportional hazard ratios (HR) for all-cause mortality

| Categories | | Model 1 | | Model 2 | | Model 3 | |
| --- | --- | --- | --- | --- | --- | --- | --- |
| TyG-BMI | Events(%) | HR (95% CI) | P-value | HR (95% CI) | P-value | HR (95% CI) | P-value |
| Q1（N=106） | 27 (25.47) | Ref |  | Ref |  | Ref |  |
| Q2（N=106） | 21 (19.81) | 0.75（0.42-1.33） | 0.32 | 0.75（0.42-1.35） | 0.34 | 0.52（0.28-0.96） | 0.037 |
| Q3（N=106） | 13 (12.26) | 0.44（0.23-0.85） | 0.014 | 0.55（0.28-1.07） | 0.079 | 0.32（0.16-0.65） | 0.002 |
| Q4（N=105） | 9 (8.57) | 0.30（0.14-0.64） | 0.002 | 0.45（0.21-0.99） | 0.047 | 0.31（0.14-0.70） | 0.005 |

CI confidence interval, TyG-BMI triglyceride glucose-body mass index, HR hazard ratio,

Model 1: Unadjusted model.

Model 2: adjusted for gender, age, race.

Model 3: adjusted for gender, age, race, platelet, high-density lipoprotein-cholesterol, low-density lipoprotein-cholesterol, alanine aminotransferase, creatinine, blood urea nitrogen, sodium, hyperlipidemia, chronic obstructive pulmonary disease, chronic kidney disease.

Table 3. Subgroup analysis of TyG-BMI index and All-cause mortality in Heart Failure patients

| subgroups | Case | Total | Q1 | Q2 | | Q3 | | Q4 | | P for Interaction |
| --- | --- | --- | --- | --- | --- | --- | --- | --- | --- | --- |
|  |  |  |  | HR(95%CI) | P value | HR(95%CI) | P value | HR(95%CI) | P value |  |
| Age,years | | | | | | | | | | |
| >68 | 69 | 220 | ref | 0.57(0.28,1.13) | 0.11 | 0.52(0.24,1.12) | 0.10 | 0.46(0.19,1.15) | 0.10 | 0.518 |
| ≤68 | 32 | 203 | ref | 1.40(0.48,4.03) | 0.54 | 0.44(0.13,1.57) | 0.21 | 0.27(0.07,1.09) | 0.065 |  |
| Sex | | | | | | | | | | |
| male | 55 | 250 | ref | 0.44(0.18,1.06) | 0.068 | 0.27(0.10,0.77) | 0.014 | 0.21(0.07,0.64) | 0.006 | 0.161 |
| female | 46 | 173 | ref | 1.34(0.63,2.86) | 0.44 | 0.71(0.30,1.68) | 0.43 | 0.47(0.17,1.31) | 0.15 |  |
| Marital status | | | | | | | | | | |
| Married | 25 | 184 | ref | 0.33(0.12,0.92) | 0.033 | 0.31(0.11,0.92) | 0.034 | 0.27(0.08,0.85) | 0.025 | 0.675 |
| others | 45 | 239 | ref | 1.30(0.65,2.60) | 0.46 | 0.54(0.23,1.25) | 0.15 | 0.32(0.12,0.86) | 0.024 |  |
| Diabetes | | | | | | | | | | |
| YES | 39 | 162 | ref | 0.51(0.16,1.58) | 0.24 | 0.39(0.13,1.12) | 0.11 | 0.28(0.09,0.86) | 0.026 | 0.947 |
| NO | 62 | 261 | ref | 0.86(0.44,1.66) | 0.64 | 0.42(0.18,1.00) | 0.050 | 0.26(0.08,0.87) | 0.028 |  |
| AF | | | | | | | | | | |
| YES | 53 | 165 | ref | 0.48(0.22,1.09) | 0.079 | 0.51(0.22,1.19) | 0.12 | 0.25(0.07,0.86) | 0.028 | 0.780 |
| NO | 48 | 258 | ref | 1.21(0.52,2.80) | 0.65 | 0.40(0.14,1.18) | 0.10 | 0.43(0.15,1.17) | 0.10 |  |
| CHD | | | | | | | | | | |
| YES | 40 | 178 | ref | 0.90(0.34,2.42) | 0.84 | 0.84(0.29,2.39) | 0.74 | 0.50(0.15,1.71) | 0.27 | 0.153 |
| NO | 61 | 245 | ref | 0.76(0.37,1.55) | 0.45 | 0.29(0.12,0.72) | 0.008 | 0.23(0.09,0.62) | 0.003 |  |
| CKD | | | | | | | | | | |
| YES | 36 | 124 | ref | 0.45(0.15,1.32) | 0.15 | 0.31(0.10,0.98) | 0.046 | 0.29(0.09,0.94) | 0.039 | 0.841 |
| NO | 65 | 300 | ref | 0.92(0.47,1.83) | 0.82 | 0.51(0.23,1.14) | 0.10 | 0.29(0.11,0.78) | 0.014 |  |
| COPD | | | | | | | | | | |
| YES | 15 | 51 | ref | 0.50(0.11,2.25) | 0.37 | 0.24(0.03,2.19) | 0.21 | 1.06(0.29,3.96) | 0.93 | 0.059 |
| NO | 86 | 372 | ref | 0.80(0.43,1.47) | 0.47 | 0.47(0.23,0.95) | 0.034 | 0.16(0.05,0.46) | <0.001 |  |
| Antiplatelet | | | | | | | | | | |
| YES | 81 | 232 | ref | 0.60(0.32,1.13) | 0.11 | 0.38(0.18,0.80) | 0.010 | 0.26(0.11,0.60) | 0.002 | 0.315 |
| NO | 20 | 103 | ref | 2.03(0.48,8.49) | 0.33 | 0.91(0.18,4.49) | 0.90 | 0.66(0.11,3.92) | 0.64 |  |
| Hyperlipidemia | | | | | | | | | | |
| YES | 57 | 219 | ref | 0.65(0.31,1.34) | 0.24 | 0.43(0.18,1.00) | 0.051 | 0.15(0.04,0.53) | 0.003 | 0.227 |
| NO | 44 | 204 | ref | 0.87(0.35,2.15) | 0.76 | 0.44(0.15,1.26) | 0.12 | 0.54(0.20,1.47) | 0.23 |  |
| ACEI/ARB | | | | | | | | | | |
| YES | 81 | 232 | ref | 0.70(0.27,1.76) | 0.45 | 0.52(0.20,1.36) | 0.18 | 0.31(0.10,1.00) | 0.051 | 0.843 |
| NO | 20 | 103 | ref | 0.83(0.40,1.71) | 0.61 | 0.42(0.16,1.06) | 0.066 | 0.31(0.11,0.84) | 0.022 |  |
| Statin | | | | | | | | | | |
| YES | 74 | 304 | ref | 0,75(0.39,1.46) | 0.40 | 0.43(0.19,0.94) | 0.036 | 0.34(0.14,0.81) | 0.015 | 0.717 |
| NO | 27 | 119 | ref | 0.75(0,25,2.30) | 0.62 | 0.46(0.14,1.54) | 0.21 | 0.22(0.05,1.04) | 0.055 |  |
| Diuretic |  | | | | | | | | | |
| YES | 75 | 315 | ref | 0.81(0,42,1.56) | 0.52 | 0.45(0,21,0.95) | 0.036 | 0.29(0,13,0.66) | 0.003 | 0.795 |
| NO | 26 | 108 | ref | 0.54(0,16,1.80) | 0.32 | 0.33(0,07,1.54) | 0.16 | 0.27(0.03,2.15) | 0.22 |  |

HR hazard ratio, CI confidence interval, CHD coronary heart disease, AF atrial fibrillation, COPD chronic obstructive pulmonary disease, CKD chronic kidney disease, ACEI/ARB angiotensin-converting enzyme inhibitor or angiotensin receptor blocker.
